# The radiologist in the fast track breast clinic: the invisible man (woman)

**DOI:** 10.1186/bcr3805

**Published:** 2015-11-05

**Authors:** Nicholas Ridley, Charlotte Jones, Nathan Coombs, Sarah Taylor

**Affiliations:** 1 Breast Unit, Great Western Hospital, Swindon, UK; 2 Department of Surgery, Great Western Hospital, Swindon, UK

## Introduction

During a visit to radiology a GP expressed surprise when she discovered that all the breast imaging and biopsies were done by radiologists. She assumed these were done by the surgical team. This led us to review the surgical letters to GPs to see if the radiologist's contribution was acknowledged.

## Methods

We reviewed 20 surgical letters from the initial fast-track appointment to GPs about patients with a proven cancer. All imaging and biopsies were performed by a consultant radiologist or radiographer.

## Results

Seventeen (85 %) letters were written by a consultant breast surgeon and three (15 %) by breast registrars. Only one (5 %) letter mentioned radiological involvement and two described the biopsies as 'we performed' giving the impression that the biopsy had been performed by the surgical team. In 17 the description of the imaging and biopsy was neutral.

All of the letters, however, were judged to be excellent in terms of information to the GP.

## Conclusion

Many medical professionals outside of the breast team are unaware of the role of the radiologist. The radiologist, despite doing all the imaging and biopsies in our clinic (which is the case in many units around the country), was essentially invisible in 19 of 20 of the letters we reviewed. We need to debate how we promote the contribution of the radiologist. This could be by reviewing the GP letter template with our surgical colleagues or by promoting the role of radiology to GPs, clinicians, medical students and the general public.

